# Supplementary material for: The incidence of deep vein thrombosis after anterior cruciate ligament reconstruction: An analysis using routine ultrasonography of 260 patients
Source: PLoS One. 2022 Dec 14;17(12):e0279136. doi: 10.1371/journal.pone.0279136 (PMC9749981; doi:10.1371/journal.pone.0279136)
Supplement: Attachment — Submitted filename: review comment 20221001.docx [file pone.0279136.s002.docx]

This manuscript is the very interesting research about incidence of DVT after ACL reconstruction. The incidence of DVT after orthopaedic surgery is a serious problem, and it is important to examine the incidence of DVT after ACL reconstruction. I think this paper is a good paper, but there is a problem that it does not confirm that DVT did not occur before surgery. The ACL injury is a traumatic injury and may have been concurrent with deep vein injury at the time of injury, and DVT may have occurred preoperatively. I consider that the high incidence of postoperative DVT in multiligament injuries is also due to the high-energy injury at the time of injury, and the possibility that DVT had already occurred before surgery cannot be ruled out. We believe it is necessary to make an accurate statement on this point. These points should be revised for this study to be relevant for publication in the PLOS ONE.

Title

For the aforementioned reasons, I consider the expression "True" to be an overstatement. Please change the title.

Introduction, Materials and Methods, Results, Figures

Well written.
