# Supplementary material for: The incidence of deep vein thrombosis after anterior cruciate ligament reconstruction: An analysis using routine ultrasonography of 260 patients
Source: PLoS One. 2022 Dec 14;17(12):e0279136. doi: 10.1371/journal.pone.0279136 (PMC9749981; doi:10.1371/journal.pone.0279136)
Supplement: Attachment — Submitted filename: Response to Reviewers Comments.docx [file pone.0279136.s003.docx]

First of all, I would like to congratulate you for this intersting article entitled 'The true incidence of deep vein thrombosis after anterior cruciate ligament reconstruction'.

The manuscript has been evaluated by three reviewers, and their comments are available below.

I also want to ask you a few questions:

- Do you routinely have ultrasound done to each of your cases after this type of surgical repair?

Ultrasonography at one week postoperatively was routinely performed during the study period. However, the postoperative protocol has now been changed based on the results of this study.

- Have you administered prophylactic anticoagulation to these patients according to your National Guidelines?

Thromboprophylaxis was not routinely applied except for anti-embolism stockings and early postoperative ambulation (Line 100–101) because the current guidelines did not advocate for routine anticoagulation in knee arthroscopy.

- Were all DVT patients or those with popliteal DVT asymptomatic?

All popliteal DVT patients had no related symptoms (Line 151).

-Why did you perform thoracic CT for pulmonary embolism (PE) in all patients with DVT? - How many patients have you detected PE? You should also add this information to the results section.

CT for pulmonary embolism was performed only for the patients with proximal DVT, which was determined after consultation with pulmonologists. There was no pulmonary embolism detected on CT scans. The manuscript has been revised accordingly (Line 108–110, 150–151).

3. In your Data Availability statement, you have not specified where the minimal data set underlying the results described in your manuscript can be found. PLOS defines a study's minimal data set as the underlying data used to reach the conclusions drawn in the manuscript and any additional data required to replicate the reported study findings in their entirety. All PLOS journals require that the minimal data set be made fully available. For more information about our data policy, please see http://journals.plos.org/plosone/s/data-availability.

Upon re-submitting your revised manuscript, please upload your study’s minimal underlying data set as either Supporting Information files or to a stable, public repository and include the relevant URLs, DOIs, or accession numbers within your revised cover letter. For a list of acceptable repositories, please see http://journals.plos.org/plosone/s/data-availability#loc-recommended-repositories. Any potentially identifying patient information must be fully anonymized.

Important: If there are ethical or legal restrictions to sharing your data publicly, please explain these restrictions in detail. Please see our guidelines for more information on what we consider unacceptable restrictions to publicly sharing data: http://journals.plos.org/plosone/s/data-availability#loc-unacceptable-data-access-restrictions. Note that it is not acceptable for the authors to be the sole named individuals responsible for ensuring data access.

We will update your Data Availability statement to reflect the information you provide in your cover letter.

Reviewers' comments:

Reviewer's Responses to Questions

Comments to the Author

1. Is the manuscript technically sound, and do the data support the conclusions?

The manuscript must describe a technically sound piece of scientific research with data that supports the conclusions. Experiments must have been conducted rigorously, with appropriate controls, replication, and sample sizes. The conclusions must be drawn appropriately based on the data presented.

Reviewer #1: Partly

Reviewer #2: Partly

Reviewer #3: Partly

2. Has the statistical analysis been performed appropriately and rigorously?

Reviewer #1: Yes

Reviewer #2: I Don't Know

Reviewer #3: Yes

3. Have the authors made all data underlying the findings in their manuscript fully available?

The PLOS Data policy requires authors to make all data underlying the findings described in their manuscript fully available without restriction, with rare exception (please refer to the Data Availability Statement in the manuscript PDF file). The data should be provided as part of the manuscript or its supporting information, or deposited to a public repository. For example, in addition to summary statistics, the data points behind means, medians and variance measures should be available. If there are restrictions on publicly sharing data—e.g. participant privacy or use of data from a third party—those must be specified.

Reviewer #1: Yes

Reviewer #2: Yes

Reviewer #3: Yes

4. Is the manuscript presented in an intelligible fashion and written in standard English?

PLOS ONE does not copyedit accepted manuscripts, so the language in submitted articles must be clear, correct, and unambiguous. Any typographical or grammatical errors should be corrected at revision, so please note any specific errors here.

Reviewer #1: Yes

Reviewer #2: Yes

Reviewer #3: Yes

5. Review Comments to the Author

Please use the space provided to explain your answers to the questions above. You may also include additional comments for the author, including concerns about dual publication, research ethics, or publication ethics. (Please upload your review as an attachment if it exceeds 20,000 characters)

Reviewer #1: The authors present a well written manuscript but their study has a number of significant flaws which make it unacceptable for publication. First and foremost, the study is based on a single timepoint ultrasound evaluation for venous thrombosis one week after ACL reconstruction. There is no clinical application or utility of this finding. Without reassessment at a later timepoint with at least clinical information, and preferably repeat ultrasound evaluation, the finding of venous thrombosis in 8.1% (6.2% distal to the popliteal vein) based on US one week after surgery is an incidental finding. Unfortunately, it raises the proverbial question, so what? This finding is not surprising or concerning and will not change practice. Ideally, in order to have a clinically relevant study worthy of publication, the patients would have been re-evaluated serially, at least at 1, 3 and 6 months after surgery, with US to determine the natural history of these otherwise incidental findings. The suspicion is that the vast majority will resolve without any clinical manifestation. There is no indication for therapeutic treatment of the distal thromboses and it is not clear if the asymptomatic popliteal vein thromboses should be treated.

Specific comments

Lines 71-3: Delete this sentence

The manuscript has been revised accordingly.

Lines 97-98: Was tourniquet used during these surgeries? If so, what was the average tourniquet time?

A tourniquet was routinely used and tourniquet time was presented instead of surgical time, as suggested by another reviewer (Line 36, 114). The average surgical time was 99.0 ± 17.1 minutes (Table 1).

Lines 106-7: Distal DVT is not considered clinically important. Without serial repeat imaging, these should be excluded.

The manuscript has been revised accordingly (Line 106–108).

Lines 107-8: CT pulmonary angiography was performed for distal DVT? Did anyone have a PE? The CT angio results should be reported.

CT for pulmonary embolism was performed only for the patients with proximal DVT, which was determined after consultation with pulmonologists. There was no pulmonary embolism detected on CT scans. The manuscript has been revised accordingly (Line 108–110, 150–151).

Lines 109-113: Why not tourniquet time?

As suggested, tourniquet time was presented instead of surgical time (Line 36, 114).

Lines 121-2: Recommend regression for proximal DVT only.

As suggested, the design of the study has been revised, focusing on proximal DVT only (Line 38, 123–125, 161–163, Table 2). Accordingly, the results and conclusion have been slightly changed (Line 44–46, 49, 161–163, 176–177, 238).

This cohort is atypical for ACL reconstruction as it is older, more male and more heavily smoking than most ACL reconstruction cohorts. This may bias the results and should be explored at length in the discussion.

The discussion has been revised accordingly (Line 219–227).

Reviewer #2: Deep vein thrombosis is an important issue that is frequently encountered after orthopedic surgical interventions. In particular, screening of the asymptomatic patient group has a very important place in this regard.

In this study, I have some questions to the authors.

1- It is stated that it is a retrospective study. Can you explain the fact that in a retrospective study, control USG was requested one week after the surgical intervention? Is it a routine practice to perform control USG on all patients?

Ultrasonography at one week postoperatively was routinely performed during the study period. However, the postoperative protocol has now been changed based on the results of this study.

2- Identification of risk factors for DVT was stated as the aim of the study. However, the data on this subject is limited in the discussion and findings section. Only one analysis was made on smoking. I think it would be appropriate to change the purpose of the study or to discuss other risk factors in the discussion section.

As suggested, the purpose of the study has been revised (Line 30, 88).

Reviewer #3: This manuscript is the very interesting research about incidence of DVT after ACL reconstruction. The incidence of DVT after orthopaedic surgery is a serious problem, and it is important to examine the incidence of DVT after ACL reconstruction. I think this paper is a good paper, but there is a problem that it does not confirm that DVT did not occur before surgery. The ACL injury is a traumatic injury and may have been concurrent with deep vein injury at the time of injury, and DVT may have occurred preoperatively. I consider that the high incidence of postoperative DVT in multiligament injuries is also due to the high-energy injury at the time of injury, and the possibility that DVT had already occurred before surgery cannot be ruled out. We believe it is necessary to make an accurate statement on this point. These points should be revised for this study to be relevant for publication in the PLOS ONE.

Title

For the aforementioned reasons, I consider the expression "True" to be an overstatement. Please change the title.

As suggested, ‘true’ was removed and the title has been changed to ‘The incidence of deep vein thrombosis after anterior cruciate ligament reconstruction: an analysis using routine ultrasonography of 260 patients’.

Introduction, Materials and Methods, Results, Figures

Well written.
