# Supplementary material for: The incidence of deep vein thrombosis after anterior cruciate ligament reconstruction: An analysis using routine ultrasonography of 260 patients
Source: PLoS One. 2022 Dec 14;17(12):e0279136. doi: 10.1371/journal.pone.0279136 (PMC9749981; doi:10.1371/journal.pone.0279136)
Supplement: Attachment — Submitted filename: Response to Reviewers Comments.docx [file pone.0279136.s004.docx]

Reviewer #4: The purpose of this paper was to assess the incidence of DVT after ACL reconstruction, regardless of symptom manifestation, by analyzing 260 patients. They reported the incidence of DVT to be 8.1%, which is higher than the previously known incidence.

1. Some values in Table 2 should be checked. For example,

a. The lower bound of CI for age = 1.0 which is the same as original exp(beta)?

b. CI for diabetes mellitus

c. Lower bound of CI for OI.

As suggested, 95% CI has been presented to two decimal places to avoid confusion. The manuscript has also been revised accordingly.

Regarding the CI of diabetes mellitus, the values have been replaced with NA (not applicable).
